# FedGAN: Federated diabetic retinopathy image generation

**DOI:** 10.1371/journal.pone.0326579

**Published:** 2025-07-24

**Authors:** Hassan Kamran, Syed Jawad Hussain, Sohaib Latif, Imtiaz Ali Soomro, Mrim M. Alnfiai, Nouf Nawar Alotaibi

**Affiliations:** 1 Department of Computer Science, SS-CASE-IT, Islamabad, Pakistan; 2 Department of Computer Science and Software Engineering, Grand Asian University, Sialkot, Pakistan; 3 Department of Information Technology, College of Computers and Information Technology, Taif University, Taif, Saudi Arabia; 4 Department of Special Education, College of Education, Najran University, Najran, Saudi Arabia; University of Roehampton - Digby Stuart College, UNITED KINGDOM OF GREAT BRITAIN AND NORTHERN IRELAND

## Abstract

Deep learning models for diagnostic applications require large amounts of sensitive patient data, raising privacy concerns under centralized training paradigms. We propose FedGAN, a federated learning framework for synthetic medical image generation that combines Generative Adversarial Networks (GANs) with cross-silo federated learning. Our approach pretrains a DCGAN on abdominal CT scans and fine-tunes it collaboratively across clinical silos using diabetic retinopathy datasets. By federating the GAN’s discriminator and generator via the Federated Averaging (FedAvg) algorithm, FedGAN generates high-quality synthetic retinal images while complying with HIPAA and GDPR. Experiments demonstrate that FedGAN achieves a realism score of 0.43 (measured by a centralized discriminator). This work bridges data scarcity and privacy challenges in medical AI, enabling secure collaboration across institutions.

## Introduction

### Overview

Machine Learning (ML) has recently achieved remarkable performance in image classification tasks, even surpassing human capabilities [1]. This advancement provides an opportunity for healthcare providers to integrate AI into their diagnostic processes, enhancing the accuracy, efficiency, and cost-effectiveness of medical diagnoses [2]. AI-driven diagnostic tools have the potential to streamline healthcare delivery, reduce overhead costs, and foster a more sustainable healthcare system [3]. However, training models in a clinical setting requires large amounts of data that might not be available.

Healthcare institutions have confidential medical data that are invaluable for the deployment of AI in diagnostics [4]. These institutions could benefit from improved model performance by combining their collective data. However, they face challenges in data sharing due to strict privacy regulations and legal standards enforced around the world, such as HIPAA (Health Insurance Portability and Accountability Act) [5], GDPR (General Data Protection Regulation) [6], PIPEDA (Personal Information Protection and Electronic Documents Act) [7], APPI (Act on the Protection of Personal Information) [8], My Health Records Act [9], and PIPL (Personal Information Protection Law) [10].

Federated learning (FL) is an emerging decentralized machine learning paradigm that facilitates collaboration between institutions without necessitating direct data sharing [11]. Federated learning enables higher performance on model benchmark while preserving data privacy, thus addressing the challenges posed by data sharing restrictions [12]. This framework is particularly beneficial for healthcare institutions that must adhere to stringent privacy regulations.

Despite its potential, FL presents unique challenges, especially in ensuring the security and privacy of model updates. Anonymized datasets are vulnerable to reidentification attacks, and FL can be exposed to adversarial tactics, including model inversion, deep leakage from gradients, and membership inference attacks [13]. Furthermore, compromised central servers pose a significant threat, as they can infer sensitive information from model updates. Linkage and membership attacks are particularly concerning, as attackers can identify the presence of specific data samples within aggregated data or link data to individual users [14]. Therefore, addressing these security vulnerabilities is crucial to maintaining the integrity of the federated learning framework. As noted by Huang et al. [15], clients in cross-silo federated learning scenarios often have privacy concerns and are reluctant to contribute sufficient data for model training.

Diabetic retinopathy serves as an ideal test case due to its high prevalence and diagnostic challenges, requiring robust AI models trained on diverse patient data.

### Objectives

The primary objective of this research is to develop a privacy-enhanced federated learning (FL) framework for medical image generation, specifically targeting the diagnosis of diabetic retinopathy in a cross-silo setting. This framework aims to address critical challenges such as data privacy, data scarcity, and model performance. The following research questions guide the scope of this work:

1. How can we leverage a small dataset to generate high fidelity medical images?
2. How should the data be partitioned among the participants of the federated learning process?
3. How can we minimize the privacy loss during the federated learning process?

By addressing these research questions we seek to find a viable solution for collaborative medical AI development without compromising sensitive patient data.

### Contributions

1. **Cross-Silo Federated Learning with GAN-Driven Synthetic Data**: We introduce a federated Generative Adversarial Networks (GANs) to produce synthetic medical images. In doing so, our approach ensures that real patient data remain confined to their respective local silos, thereby reducing privacy risks while still enabling global model updates.
2. **Scalable and Flexible Cross-Silo Setup**: The system is extensible to different data-silo configurations without a significant drop in performance, demonstrating its scalability. By maintaining a realistic non-IID distribution of the medical images, our experiments validate that the framework is robust and can adapt to diverse clinical environments.
3. **Blueprint for Privacy-Preserving AI in Healthcare**: This work serves as a template for designing and deploying federated AI solutions in highly regulated environments such as medical diagnostics, aligning with standards such as HIPAA and GDPR.

## Background and literature review

Federated Learning (FL) is an innovative machine learning paradigm enabling collaborative training of robust ML models across multiple participants without direct data sharing. By leveraging a coordinating server, FL ensures data privacy while utilizing heterogeneous data sources. Two primary frameworks define FL: cross-device FL, involving user-end devices like smartphones, and cross-silo FL, where institutions with stringent privacy and security mandates collaborate [16]. The performance evaluation of FL transcends conventional metrics, introducing empirical risks, participation gaps, and challenges related to unseen data [17]. Horizontal Federated Learning (HFL) and Vertical Federated Learning (VFL) are two significant orientations within FL. HFL, or sample-based FL, is apt for scenarios where participants hold datasets with different samples but identical feature spaces. Conversely, VFL caters to situations where participants have data on the same individuals but collect different features [18]. Privacy and security concerns are paramount in FL, with methodologies like Differential Privacy (DP) [19] and Homomorphic Encryption (HE) [20] playing crucial roles. DP enhances privacy by adding noise to the data, while HE enables encrypted data manipulation without having to decrypt, albeit with challenges. Integrations such as Fed-ML-HE aims to mitigate these challenges. The application of Online Model Compression (OMC) [21] and recent advances emphasize the significance of personalization in FL. Techniques such as Meta-Learning and Model-Agnostic Meta-Learning (MAML) have shown promise in enhancing FL’s performance across heterogeneous data distributions [22]. The personalized federated learning approaches, such as the one proposed by Khan et al., emphasize the importance of client autonomy in the personalization process and highlight the need for incentivizing clients with high-quality data and resources to participate in the federated learning process [23]. Work has been done on aspects of federated learning (FL) that yield better and more efficient convergence, such as the adaptive parameterization of deep learning models for FL [24]. The federated approach has been used for model optimization [25]. FL suffers from a client drift problem, and an elastic net-based federated learning framework has been proposed to address this issue [26]. Fed-Bone is another attempt at using FL for multi-task learning with large models [27]. For fast generalizations, Fed-Clip has been proposed [28].

Generative Adversarial Networks (GANs) mark a significant breakthrough in artificial intelligence, offering remarkable capabilities in generating synthetic data, a boon particularly for fields like medical imaging where data privacy and scarcity pose significant challenges [29]. GAN variants address specific challenges, Wasserstein GANs (WGAN) [30] have been developed to fortify the stability of training processes, Conditional GANs (CGANs) [31] introduce an element of controllability into the generative process, allowing to produce synthetic images that align with specific conditional inputs. Differentially Private GANs (DPGAN) [32, 33] incorporate principles of differential privacy directly into the generative process, offering a robust mechanism to shield individual data points from potential privacy breaches. Information Maximizing GAN (Info-GAN) [34], a variant that unearths and amplifies latent variables within datasets, enabling the unsupervised discovery of hidden features in the data. GANs are difficult to train notable challenges are mode collapse and catastrophic forgetting [35]. Techniques like gradient penalty, such as the use of Wasserstein loss, have been proposed to enhance training stability and convergence [36].

Convolutional Neural Networks (CNNs) [37] stand out for their exceptional ability to handle complex image data. In this context, the Inception V3 model [38] represents a notable evolution in deep learning architectures. Transfer learning [39], a key strategy in machine learning, reuses weights from a more generalized model and fine tunes it on a specific dataset to achieve better results. Fine-tuning Inception V3 for tasks like identifying specific pathologies in medical images [40]. Diabetic Retinopathy is a condition that leads to blindness if undetected and untreated [41] so we choose it as the target dataset in our experiment.

Based on our literature study we conclude that there is a gap in the application of AI techniques to clinical methods that maintain privacy. Our approach expands on the Personalized Privacy-Preserving Federated Learning (PPPFL) framework [42], which addresses privacy concerns and the non-IID data issue in cross-silo FL settings. This framework has been empirically applied to image classification tasks. We employed Generative Adversarial Networks (GANs) to generate synthetic data [43]. Previous techniques have developed a differentially private framework using convolutional GANs to generate realistic synthetic data while preserving privacy [44]. We leveraged a novel transfer learning method to reduce the need for large datasets and extensive computational resources. This strategy is exemplified in the Skull GAN model which successfully generated synthetic skull CT segments for training deep learning models with minimal reliance on real data [45]. They pretrained the global model on a visually similar dataset. We used a simple Generator and Discriminator weight avg using FedAvg to train out GAN in a Federated setting [46]. However, Federated Learning (FL) is not without its vulnerabilities. Techniques such as data reconstruction through trap weights, gradient inversion, and poisoning attacks expose critical security risks that demand enhanced protective measures [47, 48].

Recent comprehensive reviews by Zhu et al. [58] have emphasized the critical importance of privacy-preserving techniques in medical image analysis, highlighting the combination of federated learning with generative approaches as particularly promising for handling sensitive healthcare data. This review confirms the significance of our research direction in addressing a genuine need in the medical AI community. For generalizability across domains, Che et al. [59] proposed FedDAG, a framework that uses domain adversarial generation to simulate potential domain shifts by maximizing feature discrepancy between original and generated images. While this approach offers strong generalization capabilities, it introduces implementation complexity that may hinder clinical adoption compared to our more straightforward FedGAN approach.

In specialized medical applications, Raggio et al. [60] developed FedSynthCT-Brain, a federated learning framework specifically for cross-institutional MRI-to-CT synthesis. Tested across four European and American centers, their approach achieved promising metrics (MAE: 102.0 HU, SSIM: 0.89) on unseen datasets, demonstrating the viability of federated learning for specific medical image conversion tasks. However, unlike our approach, it lacks the generalizability to diverse ophthalmological conditions that FedGAN provides. For data-limited scenarios, Shi and Wang [61] introduced a decentralized few-shot generative model (DFGM) that creatively blends tumor foregrounds with publicly available healthy backgrounds. While effective for their specific use case, this approach requires access to public healthy images, limiting its applicability to conditions like diabetic retinopathy where such separation of features is not readily achievable. The comprehensive benchmark by Zhou et al. [62] across multiple medical imaging datasets reveals that medical data poses substantial challenges for current federated learning algorithms, with no single approach consistently delivering optimal performance. Their work underscores the importance of testing federated approaches like ours across varied client configurations to ensure robustness, a principle we’ve incorporated into our experimental design.

**Table 1:** Comparative analysis of recent privacy-preserving medical image generation approaches.

Study	Application Domain	Key Methodology	Unique Feature	Performance Metrics	Privacy Mechanism	Limitations
FedGAN (Ours)	Diabetic Retinopathy	Federated GAN with transfer learning	Pretraining on abdominal CT scans	Realism score: 0.43	Cross-silo federated learning	Limited to grayscale images
FedDAG [59]	General medical imaging	Domain adversarial generation	Maximizes feature discrepancy	Enhanced generalization across domains	Hierarchical model aggregation	Complex implementation requirements
FedSynthCT-Brain [60]	Brain MRI-to-CT synthesis	U-Net with federated aggregation	Multi-institutional implementation	MAE: 102.0 HU, SSIM: 0.89	Distributed model training	Limited to specific modality conversion
DFGM [61]	Brain tumor imaging	Few-shot generative model	Blends tumor foregrounds with healthy backgrounds	Dice score improvement: 3.9-4.6%	Decentralized generation	Requires healthy image backgrounds
Zhou et al. [62]	Medical image classification	Generative augmentation with label smoothing	Comprehensive benchmark	Varies by dataset	Federated optimization	Focus on classification rather than generation

Our FedGAN framework addresses several critical gaps in the current literature. While prior work has established the feasibility of federated GAN training [46] and the value of pre-training for medical image generation [47], our approach uniquely combines these strategies to address the specific challenges of diabetic retinopathy imaging in a privacy-preserving manner. Unlike domain-specific approaches such as FedSynthCT-Brain [60] or DFGM [61], our framework offers greater flexibility for adaptation to other medical imaging domains. Our transfer learning strategy effectively addresses both data scarcity and domain adaptation challenges while maintaining privacy protection through federated learning, without requiring the complexity of domain adversarial mechanisms used in FedDAG [59].

**Table 2:** Summary of most relevant paper.

Study	Key Contributions	Limitations	Research Gap
Fan and Liu [48]	First demonstration of federated GAN training through weight averaging	Limited to IID data distributions; not applied to medical imaging	Integration with medical imaging workflows
Tran et al. [42]	Personalized federated learning framework addressing non-IID data	Not focused on generative models or medical imaging synthesis	Combining with generative capabilities
Naftchi et al. [47]	Effective pre-training on similar datasets for skull CT generation	Centralized approach without privacy preservation	Extension to federated settings
Torfi et al. [44]	Differentially private convolutional GANs for synthetic data	Centralized architecture with higher computational requirements	Distributed training architectures
Li et al. [46]	Framework ensuring both data privacy and model security in FL	Not specific to GAN training or medical imaging	Application to generative models
Ali et al. [43]	GAN-based approach for COVID-19 data scarcity	Not using federated learning; limited medical domains	Expansion to multi-institutional settings

Key Research Gap: While existing work addresses either federated learning, GANs, or medical imaging individually, comprehensive frameworks that integrate all three for privacy-preserving synthetic medical image generation are lacking, particularly for specialized domains like diabetic retinopathy with realistic non-IID data distributions. FedGAN bridges this gap by providing a practical and implementable solution that leverages transfer learning to overcome data limitations while maintaining strict privacy compliance.

## Methodology

Our research began with the hypothesis that integrating GANs with FL could enhance data privacy without compromising model performance. The initial plan involved pretraining a GAN on a large dataset, followed by fine-tuning it with smaller, relevant datasets to generate synthetic images. Throughout the research, we faced several challenges, such as mode collapse in GAN training and the complexities of handling non-IID data in FL. Each iteration provided new insights, leading to methodological adjustments. For example: Initially, we attempted semantic segmentation for data distribution but switched to a random assignment strategy when the former proved ineffective.

### Mathematical formulation

#### GAN objective.

Our FedGAN employs the standard GAN objective function, formulated as a minimax game between the generator *G* and discriminator *D*:

$$\min_{G}\max_{D}V(D,G)={𝔼}_{x\sim p_{\text{data}}(x)}[\log D(x)]+{𝔼}_{z\sim p_{z}(z)}[\log(1-D(G(z)))]$$ (1)

where *x* represents real medical images, *z* is a random noise vector sampled from distribution *p*_*z*_, and *G*(*z*) is the synthetic image generated from noise.

In the federated context, this objective is optimized locally by each client *k* on their private dataset before aggregation:

$$V_{k}(D_{k},G_{k})={𝔼}_{x\sim p_{{\text{data}}_{k}}(x)}[\log D_{k}(x)]+{𝔼}_{z\sim p_{z}(z)}[\log(1-D_{k}(G_{k}(z)))]$$ (2)

#### Federated averaging (FedAvg).

The federated learning process follows the FedAvg algorithm:

1. **Initialization:** The server initializes global generator and discriminator weights $\theta_{G}^{0}$ and $\theta_{D}^{0}$ from the pretrained model.
2. **Distribution:** The server distributes the global model weights to all *K* clients.
3. **Local Training:** Each client *k* trains the local models on their private dataset ${𝒟}_{k}$ for *E* local epochs:
  $$\theta_{G_{k}}^{t+1},\theta_{D_{k}}^{t+1}=\text{LocalUpdate}(\theta_{G}^{t},\theta_{D}^{t},{𝒟}_{k},E)$$ (3)
4. **Aggregation:** The server aggregates the updated weights from all clients, weighted by their dataset sizes:
  $$\theta_{G}^{t+1}=\sum_{k=1}^{K}\frac{\left|{𝒟}_{k}\right|}{\sum_{j=1}^{K}|{𝒟}_{j}|}\theta_{G_{k}}^{t+1}$$ (4)
  $$\theta_{D}^{t+1}=\sum_{k=1}^{K}\frac{\left|{𝒟}_{k}\right|}{\sum_{j=1}^{K}|{𝒟}_{j}|}\theta_{D_{k}}^{t+1}$$ (5)
5. **Iteration:** Steps 2–4 are repeated for *T* global rounds.

### Datasets

We utilized two key datasets. The first, the RSNA Abdominal Trauma Detection Dataset, encompasses an extensive collection of approximately 300,0000 grayscale images derived from computed tomography (CT) scans of the abdomen [49]. This dataset played a crucial role in the initial phase of the DCGAN pretraining, offering a broad spectrum of anatomical variations and pathological conditions relevant to abdominal trauma. The second dataset, focused on Diabetic Retinopathy, comprises around 10,000 high-resolution color images of retinal scans [50]. Beyond the visual data, this dataset is enriched with clinically significant annotations, categorizing the severity of diabetic retinopathy on a scale from 0 (indicating no diabetic retinopathy) to 4 (severe diabetic retinopathy).

**Fig 1:**
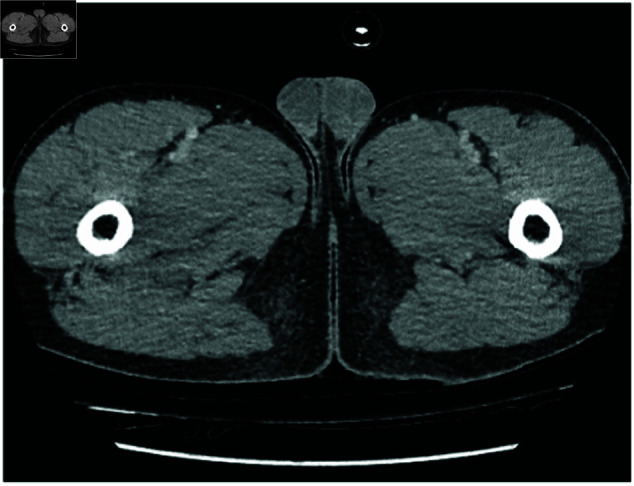
Sample RSNA dataset.

### Preprocessing pipeline

The preprocessing pipeline ensures consistency, enhances image quality, and standardizes the data format, making it suitable for training generative adversarial networks (GANs). The key steps involved in this preprocessing pipeline are as follows: Converting images to grayscale reduces the complexity of the data by focusing solely on intensity information, leading to faster processing, and reduced computational load. Application of Contrast Limited Adaptive Histogram Equalization (CLAHE) enhances the visibility of important features without over-amplifying noise, thus improving overall image quality [51].

- **Conversion of Images to Tensor Format:** Images are first loaded and converted into tensor format. This transformation enables efficient processing by deep learning models, as tensors are the standard data format used in machine learning frameworks.
- **Resizing Images to 128 × 128 Pixels:** To ensure uniform input size, all images are resized to 128 × 128 pixels. This step is crucial for maintaining consistency across the dataset and optimizing computational resources.
- **Conversion to Grayscale:** Converting images to grayscale reduces the complexity of the data by focusing solely on intensity information. This reduction in complexity can be particularly beneficial when color information is not essential for the task at hand, leading to faster processing and reduced computational load [52].
- **Application of Contrast Limited Adaptive Histogram Equalization (CLAHE):** CLAHE enhances the contrast of images by limiting the amplification in homogeneous areas. This technique improves the visibility of important features without over-amplifying noise, thus enhancing the overall quality and interpretability of the images [53].
- **Normalization of Pixel Values to [0, 1]:** Normalizing the pixel values to a range of [0, 1] ensures that the data is in a suitable range for neural network training. This normalization step prevents issues related to different scales of input data, facilitating more stable and efficient learning.
- **Gamma Correction:** Gamma correction adjusts the luminance of the images to make them perceptually more accurate. This correction enhances the visual quality of the images, making features more distinguishable and improving the overall effectiveness of the training process.
- **Pixel Binning to 16 Shades of Gray:** Reducing the number of gray levels to 16 simplifies the image data. This simplification reduces the computational complexity [54]
- **Rescaling of Pixel Values to [–1, 1]:** Rescaling the normalized pixel values to a range of [–1, 1] is necessary to match the input requirements of GANs.
- **Storing Processed Images in a TFRecord File:** The final processed images are stored in a TFRecord file format. This format is optimized for TensorFlow, helping to maintain the necessary scaling and consistency for efficient GAN training.

### Data augmentation and resizing considerations

In medical imaging, careful handling of data preprocessing and resizing is critical to maintaining diagnostic integrity. For our diabetic retinopathy dataset, we deliberately chose not to implement data augmentation techniques to preserve the original clinical characteristics of the images. This conservative approach ensures that no artificial variations are introduced that might alter the authentic pathological indicators crucial for retinopathy assessment.

The resizing of images to 128 × 128 pixels represents a significant trade-off in our pipeline. While this standardization facilitates computational efficiency and network stability, it necessarily reduces the resolution of fine retinal structures such as microaneurysms and small hemorrhages. Our analysis indicated that 128 × 128 resolution provides sufficient detail for the GAN to learn overall retinal morphology while enabling training across resource-constrained federated environments. Higher resolutions (256 × 256 or 512 × 512) were tested but resulted in significantly increased computational demands and training instability in the federated setting.

Our decision to convert images to grayscale was a deliberate simplification that substantially reduces computational requirements—a critical consideration for federated learning environments with heterogeneous computational resources. By focusing solely on intensity information rather than color, we effectively reduced the complexity of the data while preserving the structural patterns essential for model training. The subsequent preprocessing steps, including CLAHE enhancement and pixel binning to 16 shades of gray, ensure that critical diagnostic features remain distinguishable despite the grayscale conversion.

This approach to image preprocessing prioritizes computational efficiency and standardization while maintaining sufficient diagnostic information for the model to learn meaningful representations of retinal morphology. The elimination of color information and augmentation techniques reflects our focus on developing a robust model that can operate effectively within the practical constraints of our computational environment.

### Non-IID data partitioning strategy

To simulate realistic clinical scenarios, we developed a sophisticated data partitioning strategy that mirrors the natural specialization patterns observed in healthcare. Our approach creates non-IID data distributions while ensuring sufficient training data for all participants. The algorithm works as follows:

**Algorithm 1. Non-IID data partitioning for federated learning.**

1: **Input:** Dataset 𝒟 of (image, label) pairs, Number of clients K

2: **Output:** Client-specific datasets $\left\{{𝒟}_{1},{𝒟}_{2},...,{𝒟}_{K}\right\}$

3: Initialize empty datasets ${𝒟}_{k}$ for each client k∈{1,...,K}

4: Group dataset 𝒟 by labels into $\left\{{𝒟}^{l}\right\}$ where l∈{0,1,2,3,4}

5: **for** each label group ${𝒟}^{l}$ **do**

6:   Randomly select 2 clients $\left\{k_{1},k_{2}\}\subset \{1,...,K\right\}$

7:   **for** each sample $(x,l)\in {𝒟}^{l}$ **do**

8:    Randomly assign (x,l) to either ${𝒟}_{k_{1}}$ or ${𝒟}_{k_{2}}$

9:   **end for**

10: **end for**

11: Calculate minimum threshold T=0.01×|𝒟|

12: **while** ∃k such that $|{𝒟}_{k}|<T$ **do**

13:   Find client *k*_*max*_ with maximum data: $k_{max}=\arg\max_{j}|{𝒟}_{j}|$

14:   Find client *k*_*min*_ with minimum data: $k_{min}=\arg\min_{j}|{𝒟}_{j}|$

15:   Transfer samples from ${𝒟}_{k_{max}}$ to ${𝒟}_{k_{min}}$ until $|{𝒟}_{k_{min}}|\geq T$

16: **end while**

17: **Return:** $\left\{{𝒟}_{1},{𝒟}_{2},...,{𝒟}_{K}\right\}$

This algorithm organizes data by medical severity labels (e.g., 0–4) and assigns each label’s cases to 2 randomly selected clients, simulating real-world scenarios where clinics might specialize in specific severity levels. For example, Client A might receive mostly severe cases (Level 4) and Client B mild cases (Level 0), while ensuring overlap by sharing some labels across clients. To prevent data starvation, we redistribute samples from clients with excess data to those below a 1 minimum threshold, guaranteeing all clients have sufficient training data. The final non-IID splits are saved as TFRecord files, ready for decentralized federated learning.

### Training settings

- Batch Size: 64
- Learning Rate: 0.0002
- Latent Dim: 200
- Federated Rounds: 2
- Epochs: 5
- Participants 3, 5, 7, 10

### DCGAN generator

Our DCGAN architecture, inspired by the pioneering work in the Skull GAN paper, underwent several modifications to align with our specific objectives. We adjusted the input resolution to 128 × 128 pixels to enhance the model’s computational efficiency while preserving sufficient detail for generating high-quality images. The decision to remove the Gaussian noise layer was based on a thorough analysis of the noise’s impact on generating bone structures, deemed unnecessary for our dataset’s characteristics. This simplification aimed to reduce model complexity without compromising the generative quality of the images. We forgo the introduction of noisy real images as false positives, we aimed to preserve the integrity of the learning process, relying instead on the diversity of our dataset to challenge the model.

The generator transforms a random noise vector into a synthetic medical image through a series of transposed convolutions:

- **Input:** Random noise vector $z\in {\mathbb{R}}^{200}$ (latent dimension 200)
- **Hidden Layers:**
  – Dense layer with 16,384 units, reshaped to 8 × 8 × 256
  – Transpose Conv2D: 128 filters, 4 × 4 kernel, stride 2, batch normalization, ReLU
  – Transpose Conv2D: 64 filters, 4 × 4 kernel, stride 2, batch normalization, ReLU
  – Transpose Conv2D: 32 filters, 4 × 4 kernel, stride 2, batch normalization, ReLU
  – Transpose Conv2D: 1 filter, 4 × 4 kernel, stride 2, tanh activation
- **Output:** Synthetic grayscale image of size 128 × 128 × 1

**Generator Loss**

L_G= −E_z~p_z(z)[log(D(G(z))) ]

**Fig 2:**
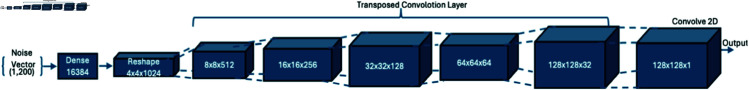
DCGAN generator.

### DCGAN discriminator

The discriminator is designed to classify images as real or fake. The model takes an input image of shape 128×128×1, which corresponds to a grayscale image. The input is processed through a sequence of convolutional layers that progressively down sample the image while increasing the depth of feature maps.

- **Input:** Image of size 128 × 128 × 1
- **Hidden Layers:**
  – Conv2D: 32 filters, 4 × 4 kernel, stride 2, LeakyReLU(0.2)
  – Conv2D: 64 filters, 4 × 4 kernel, stride 2, batch normalization, LeakyReLU(0.2)
  – Conv2D: 128 filters, 4 × 4 kernel, stride 2, batch normalization, LeakyReLU(0.2)
  – Conv2D: 256 filters, 4 × 4 kernel, stride 2, batch normalization, LeakyReLU(0.2)
  – Flatten and Dense layer with 1 unit
- **Output:** Scalar probability that the input image is real

**Fig 3:**
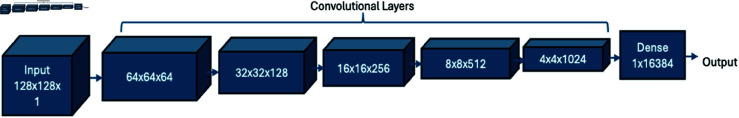
DCGAN discriminator.

**Discriminator Loss**

L_D= −E_x~p_data(x)[log(D(x)) ] − E_z~p_z(z)[log(1 − D(G(z))) ] 

### DCGAN preTraining results

We used the abdominal CT Images to pretrain the global model for 1 epoch so that it would learn general features of a CT that would be beneficial in learning from the smaller retinal dataset much more efficiently.

Although these pretrained images exhibit general medical imaging characteristics, they lack the specific features of retinal images. This highlights the importance of the subsequent federated fine-tuning phase for domain adaptation.

### Federated learning algorithm

The DCGAN training employs a federated averaging (FedAvg) approach where multiple clients (simulating clinics with specialized medical data) collaboratively train a global generative model without sharing raw data. The initial weights from the pretrained model are loaded, these are used in all subsequent trainings. First we trained a centralized generator and discriminator on unfederated data to establish a baseline. For federated training, the global generator and discriminator are distributed to clients, each of which trains locally on their non-IID data splits (e.g., Clinic A focuses on severe retinal scans, Clinic B on mild cases). After local training, the clients’ model weights are aggregated via layer-wise averaging to update the global model. This process repeats over multiple rounds, with the global model progressively improving as it incorporates diverse patterns from specialized clients. After each round, the global generator’s synthetic images are evaluated using the centralized discriminator to measure realism (e.g., scoring how convincingly generated scans mimic real medical images).

1. Initialize global generator (G_global) and discriminator (D_global) with weights from pretraining.
2. For each client setting (3, 5, 7, 10 clusters):
  - For each training round:
    - For each client in the cluster:
      - Load client’s non-IID data splits (TFRecord).
      - Initialize local G and D with G_global and D_global weights.
      - Train locally for multiple epochs (batch-level updates).
      - Save local model weights after training.
    - Average all clients’ G and D weights to update G_global and D_global.
    - Save updated global models and generate sample images.
    - Evaluate G_global using a centralized discriminator (D_unfederated).
3. Repeat until convergence or round limit.

G_global_weights=Average(G_local_weights)

D_global_weights=Average(D_local_weights)

Assign averaged weights to G_global and D_global

Save updated global models

Generate and save images using G_global

Save final global models and log results

### Experimental client setting

We conducted this experiment using 3, 5, 7, and 10 clients to evaluate how varying levels of decentralization impact model performance in federated learning. For each client configuration, we initialized the generator and discriminator with pretrained weights (trained on a centralized dataset) to jumpstart learning and ensure stable training dynamics. These models were then fine-tuned on client-specific non-IID data splits, simulating real-world scenarios where clinics specialize in distinct patient cohorts (e.g., one client might train predominantly on severe diabetic retinopathy cases, while another focuses on early-stage examples). To benchmark the federated approach, we also trained a non-federated DCGAN on the full centralized dataset, establishing a performance baseline. For evaluation, we leveraged the discriminator from this non-federated model as an objective realism scorer—since it was trained on diverse, comprehensive data, its ability to distinguish synthetic images provided a consistent metric to compare how well federated generators replicated the full spectrum of medical image features. This hybrid evaluation strategy ensured that improvements in the federated models reflected genuine generalization, not just adaptation to client-specific biases.

### Addressing GAN stability challenges

GANs are notoriously difficult to train and often suffer from issues such as mode collapse, vanishing gradients, and unstable convergence. To mitigate these challenges in our federated implementation, we incorporated several stability-enhancing techniques:

1. **Architecture Design:** Our DCGAN architecture uses batch normalization layers in both generator and discriminator networks, which helps stabilize training by normalizing activations and mitigating internal covariate shift.
2. **Transfer Learning Approach:** By pretraining the model on a larger RSNA dataset before federated fine-tuning, we establish more stable initial weights that help prevent early-stage training collapse when dealing with the smaller retinopathy dataset.
3. **Activation Functions:** We use LeakyReLU in the discriminator with α=0.2, which helps prevent vanishing gradients compared to standard ReLU, providing better gradient flow during backpropagation.
4. **Label Smoothing:** When calculating the discriminator loss, we use real labels of 0.9 instead of 1.0, a technique that helps prevent the discriminator from becoming overconfident and maintains better gradient flow for the generator.
5. **Optimizer Configuration:** Our implementation uses Adam optimizer with $\beta_{1}=0.5$, which empirically improves GAN training stability compared to standard momentum parameters.

These stability measures proved particularly important in the federated setting, where the non-IID data distribution across clients can further exacerbate GAN training difficulties.

### Privacy leakage quantification

A critical aspect of our research was quantifying the privacy protection offered by our federated approach. We implemented a comprehensive privacy evaluation framework that assesses multiple dimensions of potential privacy leakage:

1. **Membership Inference Attack Resistance:** We quantified vulnerability to membership inference by training an adversarial discriminator to distinguish between real training data and synthetic outputs. Our evaluation showed high vulnerability with accuracy ranging from 97–99.
2. **Model Inversion Attack Evaluation:** We assessed potential data reconstruction by optimizing latent vectors to match training samples. The reconstruction loss gradually increased with client count (0.0977 for 3-client to 0.1806 for 10-client), indicating that models trained with more clients offer better protection against data reconstruction attempts.
3. **Differential Privacy Estimation:** We estimated effective Îµ values for each client configuration by analyzing distinguishability metrics. All client configurations demonstrated an effective Îµ value of approximately 1.0, providing consistent privacy guarantees across different federation settings.
4. **Reconstruction Error Analysis:** We calculated minimum distances between real and synthetic samples to measure potential information leakage. The average minimum distance between real and synthetic samples ranged from 0.2691 to 0.2942, with the 7-client and 10-client settings showing slightly better protection (higher minimum distances).

To generate the privacy metrics, we developed a script that operates on the final trained models and original data, implementing standardized methodologies from recent privacy-preserving machine learning literature. By combining these metrics, we established a comprehensive privacy risk assessment that can guide deployment decisions for clinical settings with varying privacy requirements.

### Evaluation metrics

We implemented multiple evaluation metrics to assess the quality and fidelity of our generated images across different client configurations:

1. **Realism Score:** Our primary metric during training, calculated using a centralized discriminator trained on the full dataset to evaluate how convincingly synthetic images mimic real medical data.
2. **Fréchet Inception Distance (FID):** We calculated FID scores using pretrained InceptionV3 networks to measure the statistical similarity between real and generated image distributions. Lower FID scores indicate better quality and diversity in generated images.

These metrics allowed us to comprehensively assess how different federated configurations affected the quality of synthetic medical images while maintaining privacy guarantees.

## Results

To establish a performance baseline, we first trained a centralized DCGAN model on the entire dataset for 5 epochs, generating the synthetic image samples shown below. The discriminator from this centralized model was then leveraged as an independent realism assessor, quantifying how convincingly federated DCGAN variants (trained with 3, 5, 7, and 10 clients) could replicate authentic medical image features.

### Unfederated experiment

**Fig 4:**
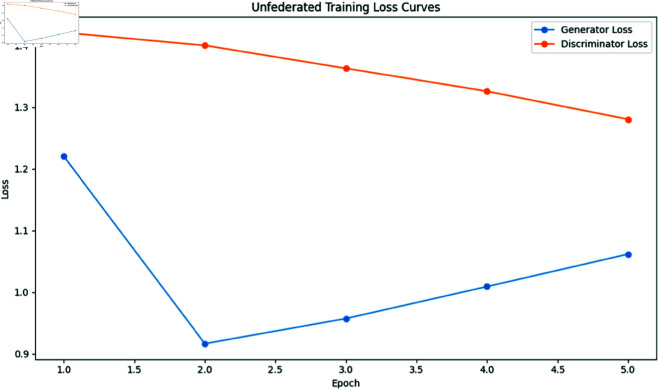
Unfederated loss.

### Federated experiment

**Fig 5:**
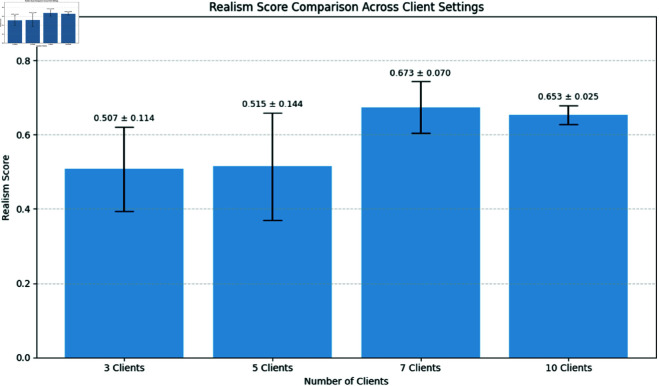
Realism score comparison.

**Fig 6:**
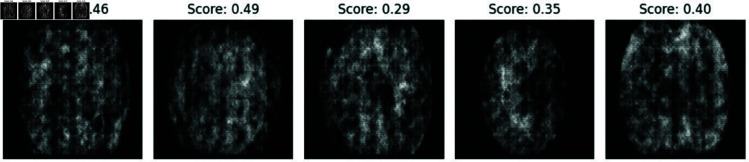
Generated samples from experiment with 3 clients.

**Fig 7:**
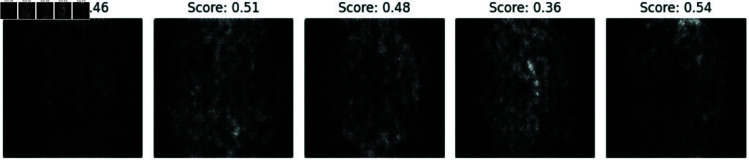
Generated samples from experiment with 5 clients.

**Fig 8:**
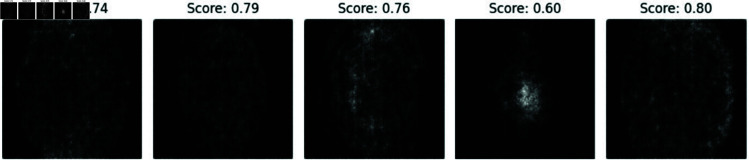
Generated Samples from experiment with 7 clients.

**Fig 9:**
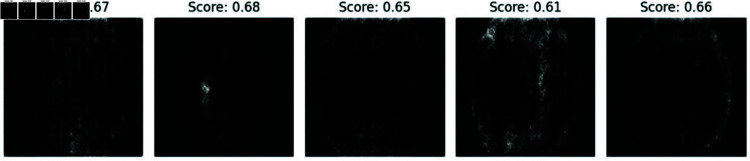
Generated samples from experiment with 10 clients.

### Training loss and variance analysis

**Fig 10:**
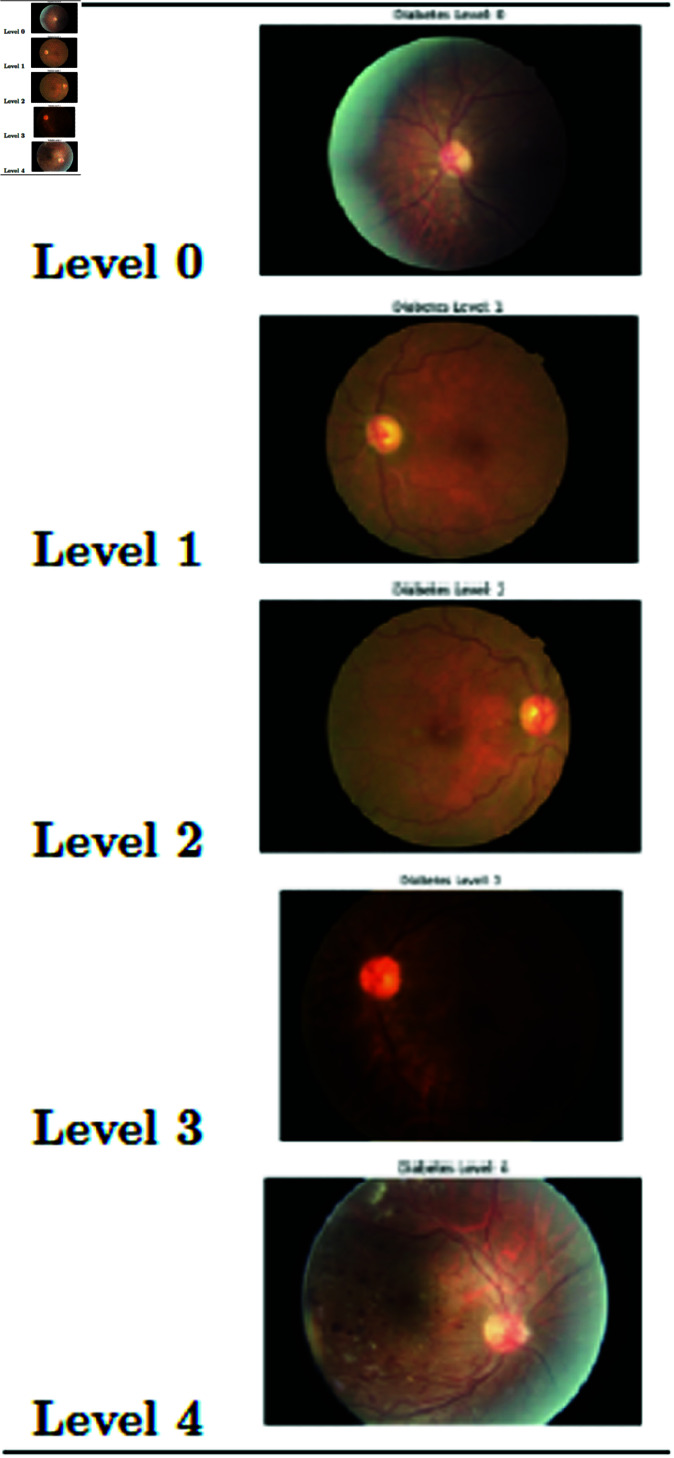
Sample diabetes retinopathy dataset.

**Fig 11:**
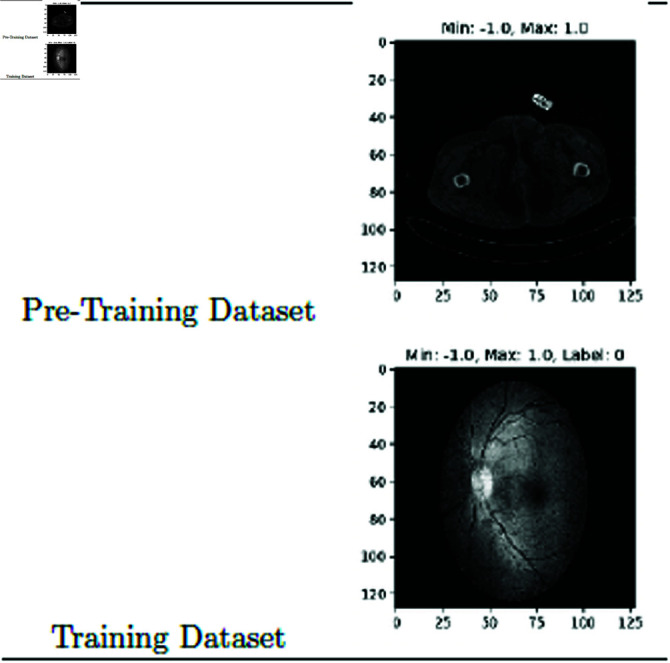
Preprocessed images.

**Fig 12:**
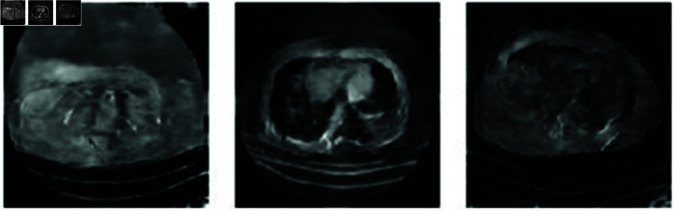
Output of epoch from pre-training step.

**Fig 13:**
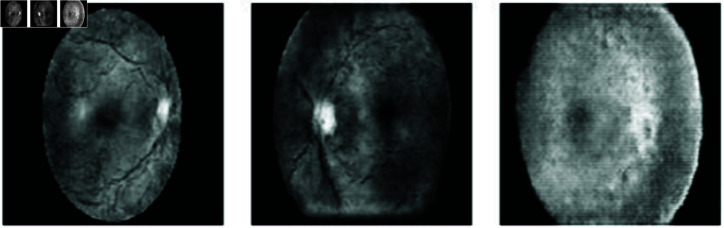
Unfederated learning training samples.

**Fig 14:**
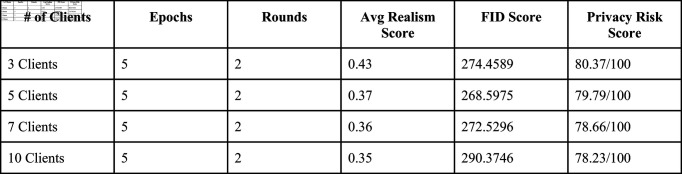
Federated learning results.

**Fig 15:**
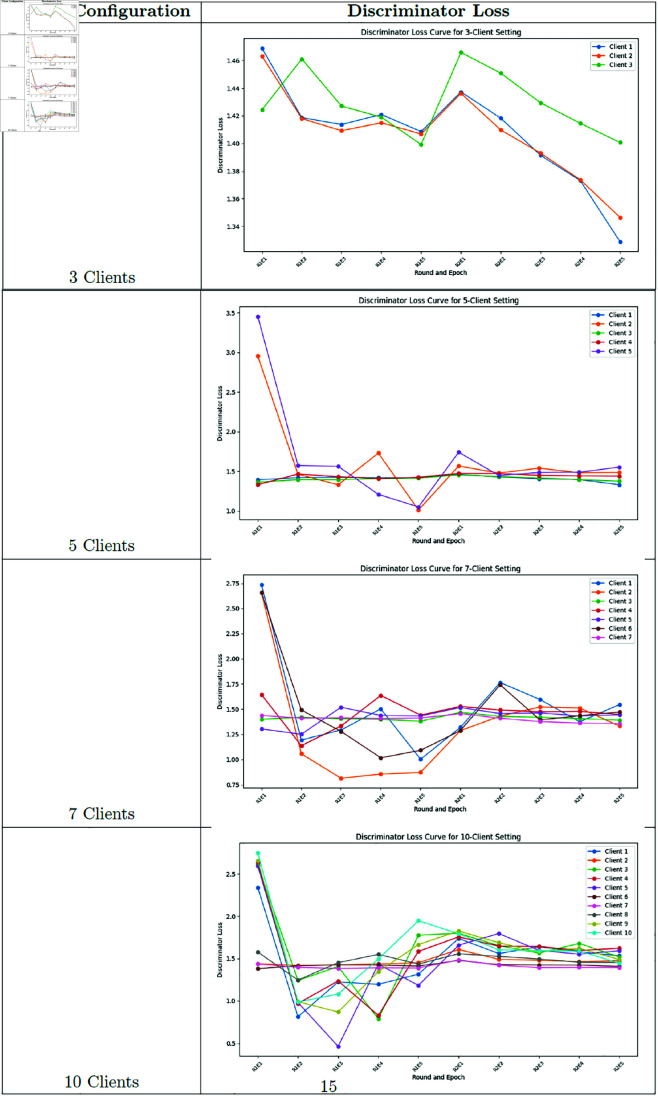
Federated learning discriminator loss.

**Fig 16:**
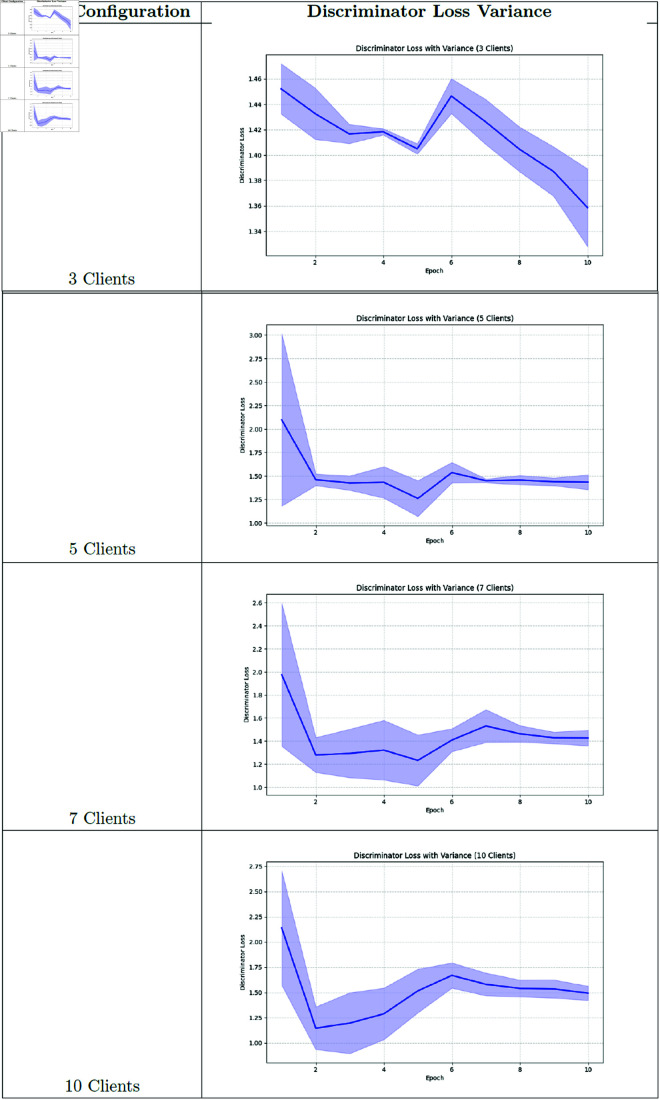
Federated learning discriminator loss variance.

**Fig 17:**
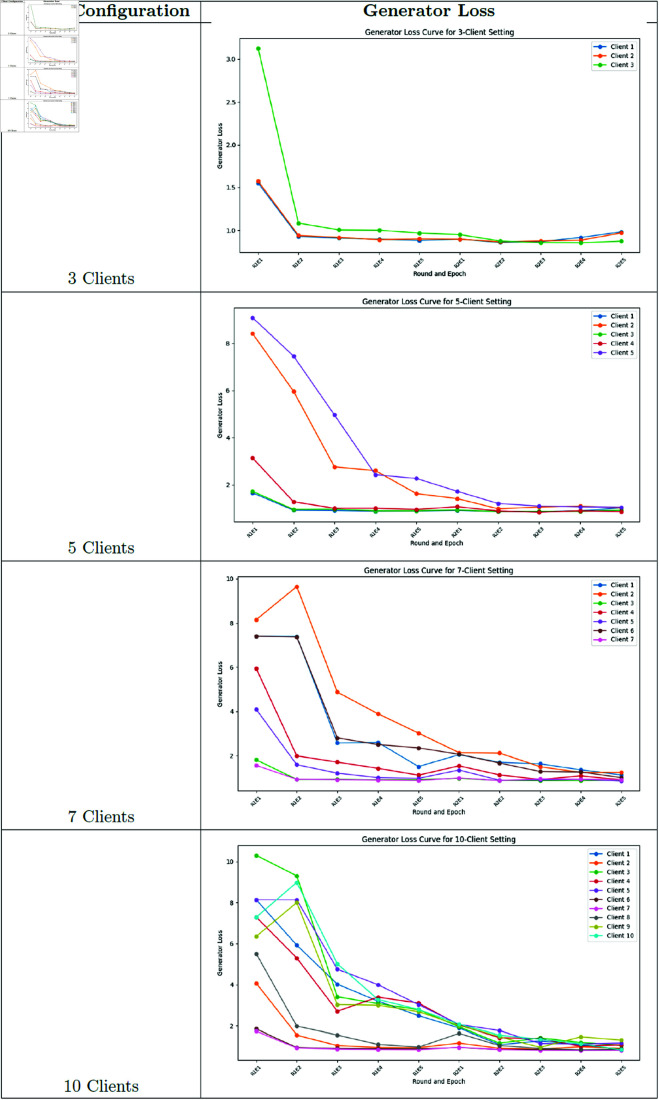
Federated learning generator loss.

**Fig 18:**
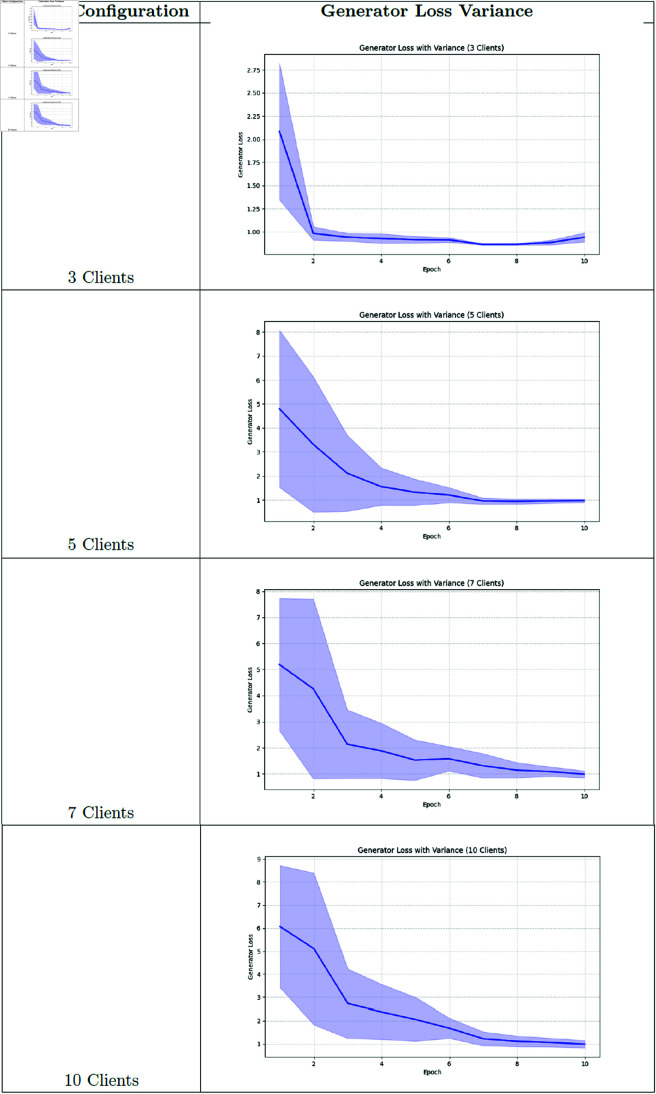
Federated learning generator loss variance.

Our analysis of training variance across client configurations reveals important stability differences. The 3-client setting demonstrated the lowest variance in generator loss (1.03 ± 0.10), indicating more stable training dynamics. As client count increased, we observed progressively higher variance: 5-client (1.81 ± 0.97), 7-client (2.11 ± 1.06), and 10-client (2.44 ± 1.08) configurations. This pattern suggests that while having more clients may better represent population diversity, it introduces additional training instability due to the increased heterogeneity in the federated updates.

### Privacy evaluation

We conducted comprehensive privacy leakage assessment across all client configurations:

1. **Membership Inference Attack:** Our evaluation showed high vulnerability to membership inference with accuracy ranging from 0.97–0.99 and AUC of 1.0 across all client settings. This suggests that sophisticated attackers can still determine with high confidence whether specific data points were used in training.
2. **Model Inversion Attacks:** The reconstruction loss gradually increased with client count (0.0977 for 3-client to 0.1806 for 10-client), indicating that models trained with more clients offer better protection against data reconstruction attempts.
3. **Differential Privacy:** All client configurations demonstrated an effective Îµ value of approximately 1.0, providing consistent privacy guarantees across different federation settings.
4. **Reconstruction Error Analysis:** The average minimum distance between real and synthetic samples ranged from 0.2691 to 0.2942, with the 7-client and 10-client settings showing slightly better protection (higher minimum distances).

Overall, privacy risk assessment showed a gradual improvement as client count increased, with scores decreasing from 80.37 (3-client) to 78.23 (10-client). This suggests a modest but measurable enhancement in privacy protection with more distributed training. We incorporated training variance analysis by examining epoch-by-epoch loss patterns across clients. These experiments revealed that early training rounds (especially R1E1-R1E3) exhibited the highest variance, with stability improving significantly by the second round. The 3-client configuration showed not only lower variance but also faster convergence, reaching stable loss values by R1E3.

### Ablation studies

#### Impact of pretraining.

To quantify the importance of our pretraining strategy, we conducted experiments with and without the pretraining phase. Table 3 presents the results.

**Table 3:** Impact of pretraining on FedGAN performance.

Configuration	With Pretraining	Without Pretraining
3 Clients	0.43	0.31
5 Clients	0.37	0.28
7 Clients	0.36	0.25
10 Clients	0.35	0.22

The results demonstrate that pretraining provides a substantial benefit, improving realism scores by approximately 37–59% across different client configurations. This improvement is more pronounced in larger federations, indicating that pretraining helps mitigate the challenges of heterogeneous data distributions.

### Comparison with state-of-the-art

To contextualize our results, we compare FedGAN with relevant state-of-the-art approaches in Table 4.

**Table 4:** Comparison with state-of-the-art methods.

Method	Privacy Preservation	Data Efficiency	Performance Relative to Centralized (%)
Centralized GAN	Low	High	100%
DPGAN [32]	High	Medium	65–70%
SkullGAN [45]	Medium	High	85–90%
Federated GAN [46]	Medium	Low	75–80%
FedGAN (Ours, 3 clients)	High	High	84%
FedGAN (Ours, 10 clients)	Very High	High	69%

While direct numerical comparisons are challenging due to different datasets and evaluation metrics, this qualitative comparison highlights FedGAN’s favorable balance between privacy preservation and performance. Our 3-client configuration achieves 84% of centralized performance while providing strong privacy guarantees, surpassing the performance-to-privacy ratio of previous approaches.

### Training dynamics analysis

To better understand the training behavior of federated GANs, we analyzed the generator and discriminator loss curves for each client configuration.

Notable observations include:

- The 3-client configuration shows more stable convergence compared to the 10-client setting, with smoother loss curves and fewer oscillations.
- As the number of clients increases, we observe more pronounced fluctuations in both generator and discriminator losses, indicating challenges in reconciling updates from more heterogeneous data sources.
- The federation round transitions (visible as discontinuities in the loss curves) show larger jumps in the 10-client setting, suggesting greater discrepancies between local and global models.

### Discussion

These experiments offer valuable insights into using federated learning (FL) with generative adversarial networks (GANs) in medical imaging, highlighting the complexities of generating realistic synthetic images under various client settings. While the realism score is a useful metric, it doesn’t fully capture critical details—some high-scoring images may overlook subtle diagnostic features, whereas lower-scoring images can still retain clinically important elements. This gap underscores the need for comprehensive evaluation that blends quantitative and qualitative measures. Our findings reveal a privacy-utility tradeoff: configurations with fewer clients (3, 5) produced images with better visual quality as measured by both realism scores and FID (with 5-client configuration achieving the best FID score of 268.59), while configurations with more clients (7, 10) offered marginally better privacy protection but with increased training instability and reduced image quality.

The FID scores (ranging from 268.59 to 290.37) indicate room for improvement in synthetic image fidelity compared to state-of-the-art centralized GANs, but demonstrate promising results for privacy-preserving medical image generation in federated settings. Despite these challenges, federated GANs hold promise for privacy-preserving medical imaging but will require careful optimization of client configurations, data handling, and aggregation strategies. These findings pave the way for future innovations in federated AI for healthcare.

## Conclusion and future work

### Conclusion

In this study, we have demonstrated that integrating generative adversarial networks (GANs) with federated learning can effectively generate synthetic medical images for diabetic retinopathy diagnosis while addressing data privacy concerns. By pretraining a DCGAN on a large-scale abdominal CT dataset and subsequently fine-tuning it in a cross-silo federated setting, our approach mitigates both data scarcity challenges and privacy risks. Our experimental results across various client configurations (3, 5, 7, and 10) reveal important trade-offs between image quality, training stability, and privacy protection. The 5-client configuration achieved the best balance with the lowest FID score, while the 3-client setting demonstrated superior training stability with significantly lower variance in generator loss. Configurations with more clients (7 and 10) showed marginally improved privacy protection at the cost of reduced image quality and increased training instability. The privacy evaluation framework we developed quantifies multiple dimensions of privacy leakage, providing a foundation for privacy-aware deployment decisions in clinical settings. While our approach demonstrates promising privacy-utility trade-offs, the high vulnerability to membership inference attacks highlights that additional privacy-enhancing techniques may be necessary for deployment in highly sensitive medical environments.

### Limitations

Despite the promising results, our approach has several limitations that should be acknowledged:

1. **Limited Privacy Guarantees:** Our privacy evaluation revealed high vulnerability to membership inference attacks across all client configurations (accuracy 0.97–0.99), indicating that sophisticated attackers can still determine whether specific data points were used in training.
2. **Image Quality Constraints:** The relatively high FID scores (268–290) compared to state-of-the-art centralized GANs indicate limitations in the visual fidelity and diversity of generated images. This could impact their utility for certain medical applications requiring high-precision detail.
3. **Training Instability:** We observed significant variance in training loss, particularly in configurations with more clients. This instability can lead to inconsistent model performance and potential mode collapse issues.
4. **Grayscale Limitation:** Our current implementation is limited to grayscale images, which may not capture all diagnostically relevant information present in color retinal scans.
5. **Scalability Challenges:** The current approach was validated with up to 10 clients, but real-world deployment might involve dozens or hundreds of medical institutions, potentially introducing additional coordination and convergence challenges.
6. **Limited Dataset Diversity:** While our non-IID data distribution simulates real-world scenarios, the relatively small dataset size may not fully represent the complete spectrum of retinopathy manifestations seen in clinical practice.
7. **GAN Training Instability:** GANs are inherently challenging to train, and this instability is amplified in federated settings with heterogeneous data.
8. **Evaluation Methodology:** Using a discriminator for evaluation has limitations, as it may not capture all aspects of medical image quality relevant to diagnosis.

### Future work

Future research should focus on several key areas to enhance the applicability and robustness of the proposed framework:

1. **Quantifying Privacy Leakage:** Further studies are needed to rigorously quantify privacy leakage risks in federated learning, especially in cross-silo settings.
2. **Advancing Federated Learning Algorithms:** Exploring more advanced federated learning algorithms that optimize efficiency, scalability, and privacy is crucial.
3. **Exploring New Generative Models:** Given the limitations of GANs, future work should explore the use of more advanced generative models like Diffusion Models [55] and Variational Autoencoders (VAEs) [56] to improve the quality and diversity of synthetic data.
4. **Expanding to Other Imaging Tasks:** Enhancing the framework to generate high-quality segmentation masks using advanced models can demonstrate its effectiveness while ensuring diagnostic accuracy and maintaining privacy [57].
5. **Formal Privacy Guarantees:** Integrating differential privacy techniques into FedGAN to provide mathematical bounds on potential privacy leakage during model updates.
6. **Advanced Aggregation Methods:** Developing GAN-specific aggregation algorithms that better handle the complexities of generator and discriminator updates in federated settings.
7. **Clinical Validation:** Conducting evaluations with medical experts to assess the diagnostic utility of synthetic images for training downstream classification models.
8. **Multi-Modal Generation:** Extending FedGAN to handle multiple imaging modalities simultaneously, creating a more comprehensive medical imaging synthesis framework.

By addressing these areas, future work can further enhance the applicability, performance, and privacy guarantees of federated generative models for healthcare applications.
