# Correction: FedGAN: Federated diabetic retinopathy image generation

**DOI:** 10.1371/journal.pone.0340003

**Published:** 2025-12-31

**Authors:** Hassan Kamran, Syed Jawad Hussain, Sohaib Latif, Imtiaz Ali Soomro, Mrim M. Alnfiai, Nouf Nawar Alotaibi

The Funding statement for this article is incorrect. The correct Funding statement is as follows: This research was funded by Taif University, Saudi Arabia, Project No. (TU-DSPP-2024-41).
